# Intra- and Intersession Repeatability of an Optical Quality and Intraocular Scattering Measurement System in Children

**DOI:** 10.1371/journal.pone.0142189

**Published:** 2015-11-04

**Authors:** Mi Tian, Huamao Miao, Yang Shen, Jian Gao, Xiaofen Mo, Xingtao Zhou

**Affiliations:** 1 Department of Ophthalmology, Eye and ENT Hospital of Fudan University, Myopia Key Laboratory of the Health Ministry, Shanghai, People's Republic of China; 2 Center of Clinical Epidemiology and Evidence-based Medicine, Fudan University, Shanghai, People's Republic of China; Rush University Medical Center, UNITED STATES

## Abstract

**Purpose:**

To evaluate intra- and intersession repeatability of objective optical quality and intraocular scattering measurements with a double-pass system in children.

**Methods:**

Forty-two eyes of 42 children were included in the study. An optical quality analysis system (OQAS) was used to measure optical quality parameters, including modulation transfer function cutoff frequency (MTF_cutoff_), Strehl ratio (SR), OQAS values (OV) at 3 different contrasts and objective scatter index (OSI). Three measurement sessions with 10-min intervals were operated by the same technician, and in each session four consecutive measurements were obtained.

**Results:**

Mean values for MTF_cutoff_, SR and OSI were 46.85 ± 7.45cpd, 0.27 ± 0.06 and 0.34 ± 0.22 respectively. 1) The intraclass correlation coefficients were ranged from 0.89 to 0.97 and coefficients of variation from 0.06 to 0.16 for all the parameters in the first session; the relative repeatability were 11.1% (MTF_cutoff_), 22.5% (SR), 10.9% (OV100%), 16.6% (OV2%), 22.4% (OV9%) and 56.3% (OSI). Similar results were found in the second and third sessions. 2) Bland-Altman analysis showed that narrow 95% confidence intervals (compared between the first and second sessions) ranged from -5.42 to 5.28 (MTF_cutoff_), -0.05 to 0.07 (SR), -0.18 to 0.18 (OV100%), -0.26 to 0.29 (OV20%), -0.33 to 0.39 (OV9%) and -0.11 to 0.09 (OSI); the comparison between any two of the three sessions showed similar results.

**Conclusion:**

Measurements of optical quality and intraocular scattering in children by the double-pass system showed good intra- and intersession repeatability. Retinal image quality is high and intraocular scattering is low in children.

## Introduction

The most commonly used method for objective optical quality assessment is wavefront aberration measuring. Recently, a newly developed system based on the double-pass technique has come into clinical use. The optical quality analysis system (OQAS)[1–2] directly computes the modulation transfer function (MTF) from the acquired double-pass retinal image through Fourier transformation; low-order aberrations are corrected in advance and the acquired retinal image contains both the information about high-order aberrations and intraocular scattering.[3] Previous studies have demonstrated that the double-pass system has good repeatability for optical quality measurements in adults.[4–6] The system provides parameters that derive from the MTF curve for retinal image quality assessment and objective scatter index for intraocular scattering evaluation. Normal value reference database of these parameters obtained from the double-pass system, which is available for clinical analysis, was also collected using the data from a population of healthy young adults.[7]

In adult patients, the double-pass system has been applied for optical quality evaluation after refractive error treatments. For example, it has mostly been used in assessing optical quality as well as intraocular scattering changes in different kinds of refractive surgeries, such as after phakic intraocular lens implantation,[8–9] and keratorefractive surgeries, including PRK, LASIK and femto-second laser small incision lenticule extraction (SMILE).[10–13] The system was also available in the optical quality evaluation after contact lenses were worn.[14–16] de Juan et al.[16] reported that after overnight contact lens wearing, optical quality worsened significantly and the scatter index increased significantly in eyes with corneal swelling. In addition, the OQAS system was found to be helpful for cataract and dry eye evaluation. The scatter index was reported to be a useful parameter to objectively grade cataracts in elder patients,[17–19] higher intraocular scattering indicates more severe lens opacity. And recently the scatter index was reported to have potential value in dry eye assessment.[20] In eyes with short tearfilm breakup time, MTF-related parameters decreased significantly after blinking, while the scatter index increased significantly. And in normal eyes, changes of these parameters during the 10-second period were not significant.

Optical quality and intraocular scattering measured using the double-pass system has been conducted in adults of different ages, and age was found to be an important impact factor. In Martínez-Roda’s study, [7] they reported high optical quality and low intraocular scattering in healthy young adults aged from 18 to 30 years. Kamiya et al.[3] found that optical quality degraded and intraocular scattering increased significantly with age in healthy subjects aged from 20 to 69 years. Saad et al.[5] compared the parameters between two different age groups that both had a corrected distance visual acuity (CDVA) of 20/20 or better. They found that optical quality was significantly higher and scattering was lower in the < 30 years group compared to the > 40 years group.

Optical quality assessment in children is also important, and many studies have investigated aberrations in pediatric patients.[21–23] The double-pass system holds promise for applications in children as another objective non-invasive method for assessing the optical quality. As in adults, the double-pass system could also be used in children for various aspects, including congenital cataract evaluation, optical quality assessment for refractive surgeries (corneal and lens related), and following contact lens use, e.g. orthokeratology lenses and rigid gas permeable contact lenses wearing. However, no previous study has tested the repeatability of the double-pass system in children. Children are always less cooperative in examinations than adults, and it is not clear if head movement or repositioning affects the accuracy of the measurements. In addition, optical quality values in healthy children should be equivalent to or better than the data acquired from young adults due to children’s transparent optical media. However, until now, no study has provided normal values of the parameters in children for clinical reference.

Subjects enrolled in the present study were healthy Chinese children from 10 to 15 years of age, with no other eye diseases except for low or moderate refractive error. We evaluated the intra- and intersession repeatability of the double-pass system measurements in these subjects to set a good foundation for future use of the double-pass system in children. In addition, we provided reference values of the optical quality parameters and the intraocular scattering index for further studies to compare with.

## Materials and Methods

### Subjects

All subjects in the present study were recruited from patients who underwent refractive examinations at the Outpatient Department of Ophthalmology, Eye and ENT Hospital of Fudan University in Shanghai, China. Criteria for inclusion in the study were as follows: CDVA of 20/20 or better, spherical error from -6.00D to +3.00D and cylindrical error equal to or less than 1.50D, non-contact IOP in the range of 10 to 21mmHg, and with Chinese ethnicity. Exclusion criteria were: a history of ocular disease (except for refractive error), previous ocular surgeries, contact lens treatment, and a history of systemic diseases that can affect eye health. Finally, 42 children (22 boys and 20 girls) with a mean age of 12.09 ± 1.76 years (range: 10 to 15 years) were included in this study, and only the right eyes were examined for repeatability evaluation. The mean spherical error of all the subjects was -1.68 ± 1.56D (range: +2.25 to -5.25D). The mean cylindrical error was -0.36 ± 0.41D (range: 0 to -1.50 D), and the mean spherical equivalent refraction was -1.87 ± 1.56D (range: +1.75 to -5.38D).

This study adhered to the tenets of the Declaration of Helsinki and was approved by the ethics committee of the Eye and ENT Hospital of Fudan University in Shanghai, China. Written informed consent was obtained from one of the subject’ s parent, and all the procedures were carried out with the subjects’ consent.

### Measurements

The spherical and cylindrical diopters were measured in each patient using the cycloplegic refraction before the optical quality examinations. The optical quality and intraocular scattering measurements were then carried out on the same day, straight after cycloplegic refraction, using an optical quality analysis system (OQAS^™^II, Visiometrics, Terrassa, Spain) by the same experienced technician, with an artificial pupil of 4.0 mm in diameter in mesopic conditions. During the measurements, spherical errors were automatically corrected by an incorporated optometer in the double-pass system, while external lens were required to correct cylindrical errors ≤ -0.50D. Each subject was instructed to sit in a comfortable position and blink once at the beginning of a single measurement, and then to blink freely during the measuring process. After each measurement, the subjects were asked to close their eyes for 2 seconds of rest. Four consecutive measurements were repeated in each session (3-minutes duration) for our intrasession repeatability assessment. In all, three sessions with a 10-minute interval between the two sessions were performed for the intersession repeatability evaluation; during this time interval, the subjects did not accept any ophthalmic examinations or visual tasks. Taking it all together, it took 30 minutes or so to finish the optical quality measurements in each subject.

### Objective Optical Quality and Intraocular Scattering Parameters

As described in previous studies,[8–13] the optical quality analysis system records point-source images projected on the retina from a light source using a laser diode of 780nm. For optical quality evaluation, a two-dimension MTF profile was calculated from the retinal image through Fourier transformation. To facilitate clinical use, several representative parameters including MTF cutoff frequency, Strehl ratio and OQAS values at different contrasts were collected from the MTF curve. MTF cutoff frequency (MTF_cutoff_) represents spatial frequency at which MTF value is 0.01. Usually the cutoff frequency of 30 cpd corresponds to a 20/20 visual acuity, and the maximum MTF_cutoff_ is no more than 60 cpd.[5] Strehl ratio (SR) is the ratio between two areas under the MTF profile, that is the aberrated eye to the ideal aberration-free eye,[5, 7] which ranges between 0 and 1.0, and larger SR indicates higher optical quality. Three OQAS values (OVs) at 100%, 20%, and 9% contrasts are calculated from spatial frequencies corresponding to 0.01, 0.05, and 0.1 MTFs, respectively. Take the OV100% for example, it is calculated as 0.01 MTF frequency (MTF_cutoff_) divided by 30 cpd; so is OV20% and OV9%. OVs are normalized values to be comparable to visual acuity;[24] however, they are independent of retinal and neural factors. For example, an OV100% of 1.0 is comparable to standard decimal visual acuity of 1.0.[12–13] Generally, higher values of MTF_cutoff_, SR and OVs indicate better optical quality.

Objective scatter index (OSI) is provided by the double-pass system as an objective index to estimate intraocular scattering. The system computes the OSI as the ratio of the amount of light of the peripheral zone (an annular area of 12 and 20 minutes) to the central zone (1 minute arc central peak) of the retinal image.[12] Usually with OSI values of less than or close to 1.0 are thought to indicate lower scattering in adults’ eyes.[7, 17]

### Statistical Analysis

The statistical analysis was performed using the software Statistical Package for the Social Sciences (SPSS) software version 20.0 (SPSS, Chicago, IL, USA) and MedCalc statistical software version 11.4.2.0 (MedCalc Software, Inc, Mariakerke, Belgium). Normal distributions of all the parameters were confirmed through the Kolmogorov-Smirnov test, and parametric statistics were applied for further analysis. Intrasession repeatability for measurements was assessed by means of repeated measures analysis of variance (ANOVA), and statistical parameters including the intraclass correlation coefficient (ICC), the coefficient of variation (CV), and the coefficient of reproducibility (CR). Intersession repeatability was assessed by both ANOVA and Bland-Altman analysis. A *P* value less than 0.05 was considered of statistically significant.

The ICC was used as a measure of the relative homogeneity between the repeated measurements within sessions.[24–25] Higher ICC values represent greater repeatability within repeated measurements, when there is no variance, the value will reach 1.0.[6] According to Lackner’s study,[26] the intrasession repeatability is considered to be low when the ICC value is less than 0.75, to be moderate when the value is between 0.75 and 0.90, and to be high when the value is greater than 0.90. The CV is a ratio of the standard deviation over the mean. The smaller the CV value, the lower the intrasession variation and the better the intrasession repeatability.

The coefficient of repeatability (CR) was calculated using the within-session standard deviation (S_w_) of 1.96×√(2S^2^_w_) or 2.77S_w._[25] The relative repeatability (RR) was defined as the ratio of the CR to the mean value of the measurements, which allows our results to be compared with those of other studies;[27] a lower RR represents greater intrasession repeatability. Usually an RR of less than 50% is acceptable when measuring individual eyes in biological metrics.[28]

Intersession repeatability between any two of the three sessions was assessed by Bland-Altman plots analysis, using the mean values of the four measurements of each session. Bland-Altman charts plotted the mean difference (Md) and 95% confidence intervals (CI) calculated as Md ± 1.96SD, providing an interval within which 95% of the differences between the measurement of the two sessions was supposed to fall.[29]

## Results

The measured parameters of the right eyes in all the subjects were normally distributed (*P* > 0.05). Mean values of all the measurements from the three sessions were 46.85 ± 7.45cpd for MTF_cutoff_, 0.27 ± 0.06 for SR, 1.56 ± 0.25 for OV100%, 1.65 ± 0.37 for OV20%, 1.67 ± 0.45 for OV9% and 0.34 ± 0.22 for OSI. The means and standard deviation (SD) of the optical quality parameters and intraocular scattering index in each of the three sessions are provided in Table 1.

**Table 1:** Mean and SD for the parameters in each of the three sessions.

	First session		Second session		Third session	
Parameters	Mean	SD	Mean	SD	Mean	SD
**MTF**_**cutoff**_ **(cpd)**	46.86	7.87	46.93	7.52	47.07	7.49
**SR**	0.28	0.06	0.27	0.06	0.27	0.06
**OV100%**	1.56	0.26	1.56	0.25	1.57	0.25
**OV20%**	1.69	0.37	1.68	0.36	1.69	0.35
**OV9%**	1.74	0.41	1.71	0.42	1.73	0.41
**OSI**	0.32	0.21	0.33	0.21	0.33	0.21

SD = standard deviation; MTF_cutoff_ = modulation transfer function cutoff frequency; cpd = cycles per degree; SR = strehl ratio in 2 dimensions; OV = OQAS values; OSI = objective scatter index.

In any parameter of the three sessions, no statistically significant difference was found among four consecutive measurements when the repeated-measures ANOVA was conducted (*P* > 0.05). Table 2 gives the intrasession repeatability outcomes for the measured parameters in each of the three sessions. In the first session, ICC values in most parameters were larger than 0.90 (0.93 to 0.97) except for OV9% (0.89); CV values were lower than 0.10 (0.06 to 0.10) in all the parameters except for OSI (0.16); The CRs obtained from the first session were 5.18cpd (MTF_cutoff_), 0.06 (SR), 0.17 (OV10%), 0.28 (OV2%), 0.39 (OV9%) and 0.18 (OSI). The RR was calculated as the ratio of the CR to the mean value of the measurements, and in the first session, the percentages were generally less than 50% (10.9% to 22.5%) except for the value for OSI (56.3%). These intrasession repeatability parameters (ICC, CV, CR, and RR) were similar to each other across the three sessions.

**Table 2:** Intrasession repeatability outcomes for the measurements in the three sessions.

	First session				Second session				Third session			
Parameters	ICC	CV	CR	RR(%)	ICC	CV	CR	RR(%)	ICC	CV	CR	RR(%)
**MTF**_**cutoff**_ **(cpd)**	0.97	0.06	5.18	11.1	0.96	0.06	5.19	11.1	0.95	0.06	6.06	12.9
**SR**	0.89	0.10	0.06	22.5	0.92	0.06	0.06	21.2	0.83	0.12	0.09	31.4
**OV100%**	0.97	0.06	0.17	10.9	0.96	0.10	0.17	10.9	0.95	0.06	0.20	12.7
**OV20%**	0.95	0.08	0.28	16.6	0.95	0.08	0.27	16.1	0.94	0.08	0.32	18.9
**OV9%**	0.93	0.10	0.39	22.4	0.93	0.10	0.39	22.8	0.89	0.12	0.48	27.7
**OSI**	0.97	0.16	0.18	56.3	0.98	0.16	0.14	42.4	0.98	0.18	0.14	42.4

ICC = intraclass correlation coefficient; CV = coefficient of variation; CR = coefficient of reproducibility; RR = Relative repeatability.

**Fig 1:**
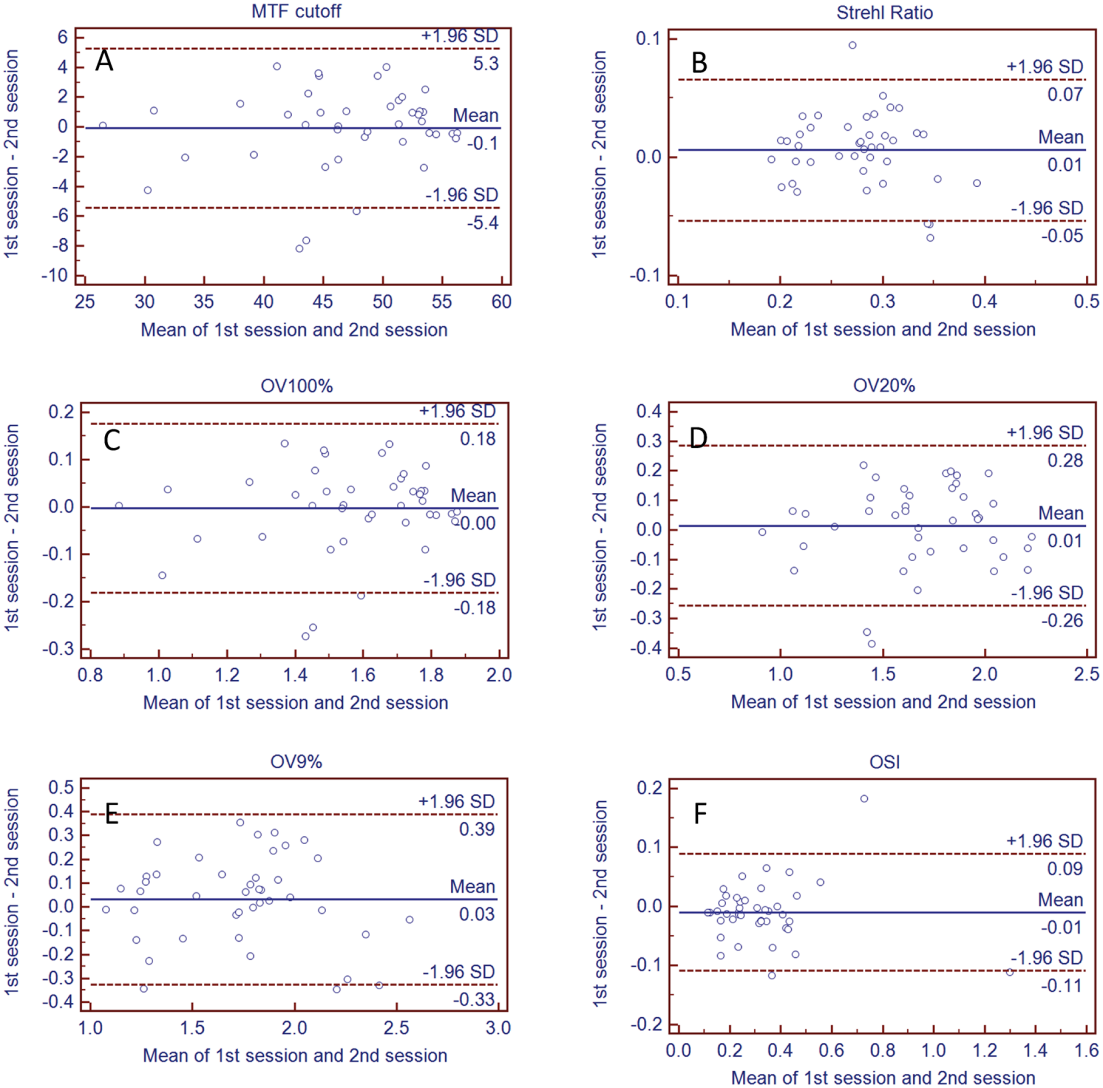
Bland-Altman plots of all parameters between the first and second session. Bland-Altman plots of the mean difference (Md) and 95% confidence intervals (CI) for MTF_cutoff_ (A), SR (B), OV100% (C), OV20% (D), OV9% (E) and OSI (F) between the first and second session.

When comparing mean values of the parameters between sessions using a repeated measures ANOVA, no statistically significant difference was found for any parameter (*P* > 0.05). Bland-Altman plots for the six parameters are shown in Fig 1, in which the comparison between the first and second sessions were conducted; the mean differences (Md) were small (close to 0), as was the range of 95% confidence intervals (CI). Similar results as our Bland-Altman analysis for intersession repeatability evaluation between the sessions were also found when comparing between the first and third sessions and also the second and third sessions. Detailed information regarding the Md and the 95% CI of each parameter in the three intersession comparisons is listed in Table 3.

**Table 3:** Bland and Altman analysis for intersession repeatability between sessions.

	1^st^ and 2^nd^ sessions		1^st^ and 3^rd^ sessions		2^nd^ and 3^rd^ sessions	
Parameters	Md	95% CI	Md	95% CI	Md	95% CI
MTF_cutoff_(cpd)	-0.07	-5.42 to 5.28	-0.21	-5.25 to 4.83	-0.14	-4.68 to 4.41
SR	0.01	-0.05 to 0.07	0.01	-0.06 to 0.07	0.00	-0.05 to 0.05
OV100%	0.00	-0.18 to 0.18	-0.01	-0.18 to 0.16	-0.01	-0.15 to 0.15
OV20%	0.01	-0.26 to 0.29	0.00	-0.27 to 0.27	-0.02	-0.24 to 0.21
OV9%	0.03	-0.33 to 0.39	0.01	-0.38 to 0.41	-0.02	-0.34 to 0.30
OSI	-0.01	-0.11 to 0.09	-0.01	-0.11 to 0.10	0.00	-0.07 to 0.08

Md = mean difference; 95% CI = 95% confidence intervals.

## Discussion

A comprehensive evaluation of optical quality needs to take into consideration both the aberration and the scattering.[5, 30] Aside from an aberrometer, the double-pass system applied in the present study is another convenient and objective method for optical quality assessment, which take into consideration both aberrations and intraocular scattering. Its repeatability and clinical value in adults was confirmed in previous studies. We suggest that the system could also be a useful method for objective optical quality evaluation in children. The present study evaluated the intra- and intersession repeatability of optical quality and intraocular scattering measurements and also the distribution of the parameters using the double-pass system in a Chinese children group aged from 10 to 15 years of age.

Optical quality has been demonstrated to decrease with advancing age due to less transparency of the refractive medium.[3] In our study population, optical quality values are high and intraocular scattering are low, suggesting a high transparency and uniformity of refractive media in children. The mean MTF_cutoff_ of 46.85cpd and the mean OSI of 0.34 in the present study were close to those reported by three other studies conducted in young healthy adults aged from 18 to 30 years. The mean MTF_cutoff_ was about 46.00cpd, and the mean OSI was 0.32 in 10 adults (23.1 ± 3.5 years, 20 to 30 years) in Vilaseca’s report; Martinez-Roda et al.[7] found the mean MTF_cutoff_ to be 44.54cpd, while the mean OSI was 0.38 in 181 adults (22.47 ± 3.04 years, 18 to 30 years). Finally, the mean MTF_cutoff_ was 39.44cpd and the mean OSI was 0.47 in 8 adults (27.5 ± 2.8 years, < 30 years) in Saad’s study.[5] These combined findings suggested that both children and young adults (≤ 30 years) have a high optical quality and low ocular scattering; their optical quality values were much better than those reported in older subjects. Saad et al.[5] determined a mean MTF_cutoff_ of 26.07cpd and a mean OSI of 1.73 in elder adults (53.1 ± 6.9 years, > 30 years) with a CDVA ≥ 20/20. Kamiya et al.[3] found a negative correlation between age and optical quality parameters and a positive correlation between age and intraocular scattering in 100 adults across a wide range of ages (44.6 ± 15.5 years, 20 to 69 years), and their mean MTF_cutoff_ was 27.76 ± 8.41cpd, while the mean OSI was 1.29 ± 0.76. A similar optical quality and scattering in children and young adults indicated that the optical quality remains stable up until the age of 30 years. Our results also add the data of healthy children to the objective optical quality and scattering database.

In any of the three sessions, no statistically significant difference was found among four consecutive measurements, indicating that the variation of the measurements did not increase over time. In general, an ICC value above 0.75 is considered to suggest good intrasession repeatability; however, most clinical applications require an ICC value of 0.90 or higher.[31] In our study, most of the ICC values of the optical quality parameters were above 0.90 and only a few were lower than but close to 0.90, such as the ICC for SR in the first and third sessions (0.89 and 0.83, respectively) and the OV9% in the third session (0.89), suggesting high intrasession repeatability of optical quality measurements in children provided by the double-pass system. ICCs in our study were much higher than those found in Tomas’s study,[6] which reported ICC values of 0.75 for MTF_cutoff_ and 0.63 for SR in adults aged from 20 to 60 years. As previously discussed, elder adults have a much lower optical quality than children and young adults, so the wide age range in Tomas’s study should be considered the main reason for their lower ICC values compared to ours. CV values of all the parameters except for OSI (0.16 to 0.18) were less than or close to 0.10 in the three sessions, which also suggested relatively small intrasession variations of the consecutive measurements. The CR values of all parameters obtained from each of the three sessions were similar. Usually an RR value of less than 50% is thought to be acceptable. All RR percentages in the present study were lower than 50% except for the OSI in the first session (56.3%). These values are also comparable to the repeatability data in Vilaseca’s study,[4] which were gathered from 10 healthy young adults. The best RR values in their study were 9.8% for MTF_cutoff_ and OV100% (examples were taken from their first session), and then OV20% (12.9%), OV9% (16.4%) and SR (17.8%). A high RR of OSI (31.4% to 40.6%) was also found in their study. In general, the ICC, CV, and RR values confirmed that the double-pass system had a high intrasession repeatability for the measurements of optical quality and intraocular scattering in children.

The three OVs are collected from the same MTF curve at different frequencies, and lower contrasts correspond to lower frequencies. Specifically, OV100% is directly calculated by dividing MTF_cutoff_ by 30, so they exhibited the same highest repeatability level. Despite the fact that OV20% and OV9% also showed good repeatability, there was a small but obvious tendency that the ICC decreased and the CV or RR increased, which means the measurement variability increased and the repeatability declined with decreasing contrast. The lowest ICC values were found in SR, which also had relative larger CV values and a lower RR compared to other parameters. OSI has the highest ICC values, which suggests a high intrasession correlation in intraocular scattering measurements; however, it also has the largest CV values and RR. Although SR and OSI exhibited worse repeatability results, they were still within the acceptable range. For instrumental measurements, the smaller the absolute value of a parameter, the greater the relative repeatability error. The mean values of SR (0.28) and OSI (0.32) were close to zero in our study, which might have contributed to their lower repeatability results compared to other parameters.

No significant difference was found in comparing mean values between sessions, indicating that the measurements of optical quality were repeatable over time in children. Bland-Altman analysis showed that mean differences between sessions were close to zero, and the range of the 95% CI was narrow; Bland-Altman plots also revealed that the differences of the six parameters was spread around the mean difference. The good intersession repeatability results suggested that neither repositioning of the subjects nor realignment of the eyes affected the measurements, which is comparable with the previous study results reported in young adults.[4]

In conclusion, measurements of optical quality and intraocular scattering using the double-pass system in children have a good intra- and intersession repeatability. In addition, as expected, retinal image quality was high and the scattering of light was low in healthy Chinese children. Our study set a good foundation for future study and clinical application of the objective and quantitative method for optical quality evaluation in children. As has been determined in adults, this non-invasive optical quality analysis system could also be suggested for clinical applications in children for various aspects, such as refractive surgeries, contact lens use and pediatric cataracts.
